# Molecular Dynamics Study on Mechanical Properties of Interface between Urea-Formaldehyde Resin and Calcium-Silicate-Hydrates

**DOI:** 10.3390/ma13184054

**Published:** 2020-09-12

**Authors:** Xianfeng Wang, Wei Xie, Taoran Li, Jun Ren, Jihua Zhu, Ningxu Han, Feng Xing

**Affiliations:** 1 Guangdong Provincial Key Laboratory of Durability for Marine Civil Engineering, College of Civil and Transportation Engineering, Shenzhen University, Shenzhen 518060, China; xfw@szu.edu.cn (X.W.); xw13671400570@163.com (W.X.); litaoran2016@email.szu.edu.cn (T.L.); nxhan@szu.edu.cn (N.H.); xingf@szu.edu.cn (F.X.); 2 School of Science, Harbin Institute of Technology, Shenzhen 518055, China

**Keywords:** calcium–silicate–hydrates, urea-formaldehyde microcapsules, molecular dynamics simulation, stress-strain, binding energy

## Abstract

Microcapsule based self-healing concrete can automatically repair damage and improve the durability of concrete structures, the performance of which depends on the binding behavior between the microcapsule wall and cement matrix. However, conventional experimental methods could not provide detailed information on a microscopic level. In this paper, through molecular dynamics simulation, three composite models of Tobermorite (Tobermorite 9 Å, Tobermorite 11 Å, Tobermorite 14 Å), a mineral similar to Calcium-Silicate–Hydrate (C–S–H) gel, with the linear urea–formaldehyde (UF), the shell of the microcapsule, were established to investigate the mechanical properties and interface binding behaviour of the Tobermorite/UF composite. The results showed that the Young’s modulus, shear modulus and bulk modulus of Tobermorite/UF were lower than that of ‘pure’ Tobermorite, whereas the tensile strength and failure strain of Tobermorite/UF were higher than that of ‘pure’ Tobermorite. Moreover, through radial distribution function (RDF) analysis, the connection between Tobermorite and UF found a strong interaction between Ca, N, and O, whereas Si from Tobermorite and N from UF did not contribute to the interface binding strength. Finally, high binding energy between the Tobermorite and UF was observed. The research results should provide insights into the interface behavior between the microcapsule wall and the cement matrix.

## 1. Introduction

The micro-cracks generated within concrete can seriously impact the performance and durability of concrete, leading to the short service life of concrete [1,2]. In recent decades, to improve the durability of concrete, many researchers have attempted to apply different technologies to prevent and repair the cracks [3,4,5]. According to the bionics principle, an advanced self-healing technology incorporating the remediation/repair agent into concrete has been developed, which can automatically locate and repair the micro-cracks and imply the self-remediation and regeneration function [6]. When the crack occurs, the repair agent embedded in the cementitious matrix can be triggered due to the change in environment, such as force, heat or chemicals, which then release the healing agent, i.e., epoxy, to cement the crack surface and delay the propagation of the cracks to achieve the self-healing target [7]. Compared to passive repair work, self-healing technology has many advantages, such as low cost, sensitive response, and accurate repair [8,9]. Among the different type of self-healing system, microcapsule-based self-healing concrete has been proven to be a good method for repairing the concrete cracks [10].

Microencapsulation is a technology for preparing core-shell structured microcapsules with a repair agent core and polymeric shell, which has been widely employed in the pharmaceutical, food and printing industries [11]. In the construction industry, the incorporation of microcapsules in concrete has been demonstrated to play an obvious and positive role for the maintenance of the mechanical properties of concrete by repairing micro-cracks [12]. This is particularly true in the case of epoxy/urea-formaldehyde (UF) microcapsules, which have attracted increased attention from both scientists and engineers [13]. In general, when the micro-cracks occur in the matrix, under the force from the tip, the UF shell of microcapsules is ruptured to release a healing agent to prevent the extension of internal micro-cracks in the structure [9]. Therefore, the addition of microcapsules in concrete could not only accurately locate the defects within the cementitious matrix, but also repair the micro-cracks [14]. To ensure the implementation of the self-healing function, it has to provide sufficient strength, so that the microcapsules should remain intact during mixing, and have the ability to be ruptured under external force when the micro-cracks occur [15]. Thus, a high adhesion strength between the microcapsule wall and the cementitious matrix is required [15,16]. Therefore, the interface properties between the shell of microcapsules and the cementitious matrix are important to achieve the self-healing performance of a microcapsule-based system. However, when confined to the limitations of current techniques, it is difficult to experimentally investigate the interface property, particularly investigation at a microscopic level.

As it is a powerful tool, molecular dynamics (MD) simulation has been applied in cementitious materials on the molecular scale [17,18,19]. It is a kind of classical mechanical model based on molecules, which is often applied to study the structure and properties of molecular systems by solving Newton’s equations of motion. Therefore, it has been widely used in the chemical industry, biomedicine and other fields [20,21]. Since the calcium–silicate–hydrates (C–S–H) gel accounts for over 60% of the total hydration products of cement, which mainly contributes to the strength of hardened cement [22,23,24], the MD simulation has been used to study the C–S–H with different proposed C–S–H models [25,26,27,28], i.e., the colloidal gel-like structure [29], the incomplete lamellar mixture of Tobermorite and Jennite [30]. Ching et al. [31] analyzed the interface of the model of an explicitly solvated mixture of C–S–H and a silicon binding peptide, and found that a strong covalent bond was formed between the composite hybrid models. Rouhi and Atfi [32] studied the interactions between graphene-like monolayers and four different polymer chains, and found that increasing repeat units increased the interaction energy between polymer and nanosheet. Alkhateb et al. [33] established the model of C–S–H/graphene composite, and calculated the interface strength between the C–S–H and the graphene. Du et al. [34] established an interface model to study the adsorption energy of the interface between an epoxy resin/C–S–H in nanoscale and reported that the Ca^2+^ ions in C–S–H migrated to the interface and formed an electrostatic bond with the hydroxide in the resin, which is the main source of adsorption energy. All the studies mentioned above have proved the feasibility of MD simulation in characterizing the interface properties between the polymeric additives and cementitious matrix, which provides potential to apply the MD simulations in investigating the interface properties between the microcapsule and the cementitious matrix. Unfortunately, the MD simulation on investigating the interface property of the composite model of cement matrix and microcapsule wall (urea–formaldehyde resin), which is of great significance in revealing the trigger mechanism in the self-healing cementitious material, has not been systematically conducted yet.

The purpose of this study is, therefore, to study the binding behavior between the Tobermorite, a mineral with similar structure of C–S–H [35,36,37,38], and the linear UF, the shell of the microcapsule, via MD simulation. Four individual models of three different types of Tobermorite and one linear UF were first built. Then, three models of a Tobermorite/UF composite were established by combining these individual models. Then, the mechanical properties, including the modulus, atomic radial distribution function (RDF), stress–strain relationship and the binding energy, were obtained via MD by uniaxially stretching the entire structure along the *z*-direction.

## 2. Computational Methodology

### 2.1. Forcite

COMPASS is an ab initio forcefield inherited from CFF (consistent force fields), a previous generation of forcefield. It has been proven that the COMPASS exhibited a good consistency with the experiment data, which therefore, means the COMPASS is often employed in the calculation of cementitious material [39,40,41]. On the other hand, CVFF, a generalized force field, has been proven to be an appropriate tool to calculate the small organic crystals and meteorological structures for calculating the structure and binding energy, due to its advantage in reasonably predicting the vibration frequency and conformational energy [39]. Therefore, in this study, COMPASS was used for the calculation of Tobermorite, a similar structure of hydrated calcium silicate (C–S–H) gel, and CVFF was applied for the simulation of urea-formaldehyde resin as the microcapsule shell.

### 2.2. Model Construction

**Figure 1:**
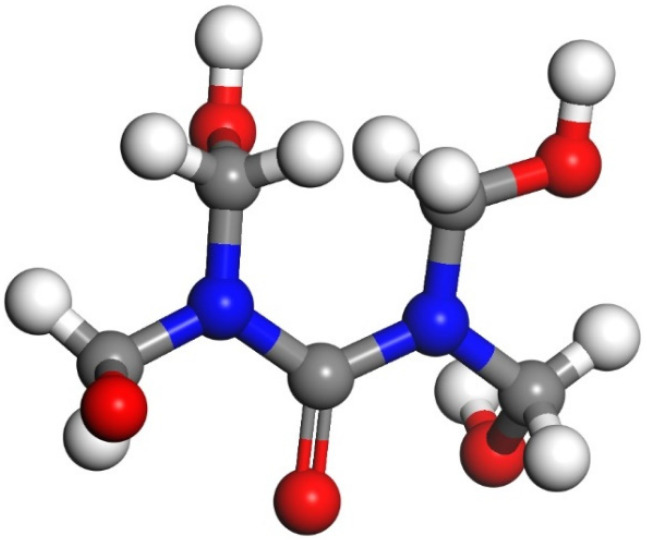
The monomer structure model of urea–formaldehyde (UF) resin (Color legend: hydrogen H (white); oxygen O (red); nitrogen N (blue) carbon C (grey)).

**Figure 2:**
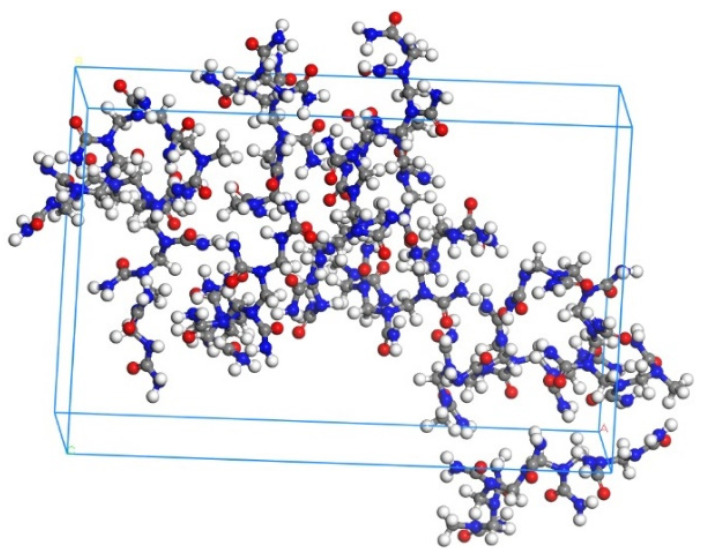
Molecular structure of UF (Color legend: hydrogen H (white); oxygen O (red); nitrogen N (blue) carbon C (grey)).

UF resin is a linear structure formed by the reaction of formaldehyde and urea under acidic conditions [42]. The chemical formula of UF resin is C_2_N_2_OH_4n_, and the monomer structure was built by following Xie’s approach (Figure 1), where the n value of the UF is usually in the range 7–10 [43]. In this study, to simplify the scenario, n = 7 was applied. Therefore, the UF chain composed of 7 repeating monomer units was built. Then, the amorphous cell of UF was constructed by an Amorphous Cell Construction. As shown in Figure 2, the amorphous cell of UF contained ten UF chains with a density of 1, and the cell lengths a and b will correspond with those of Tobermorite.

**Figure 3:**
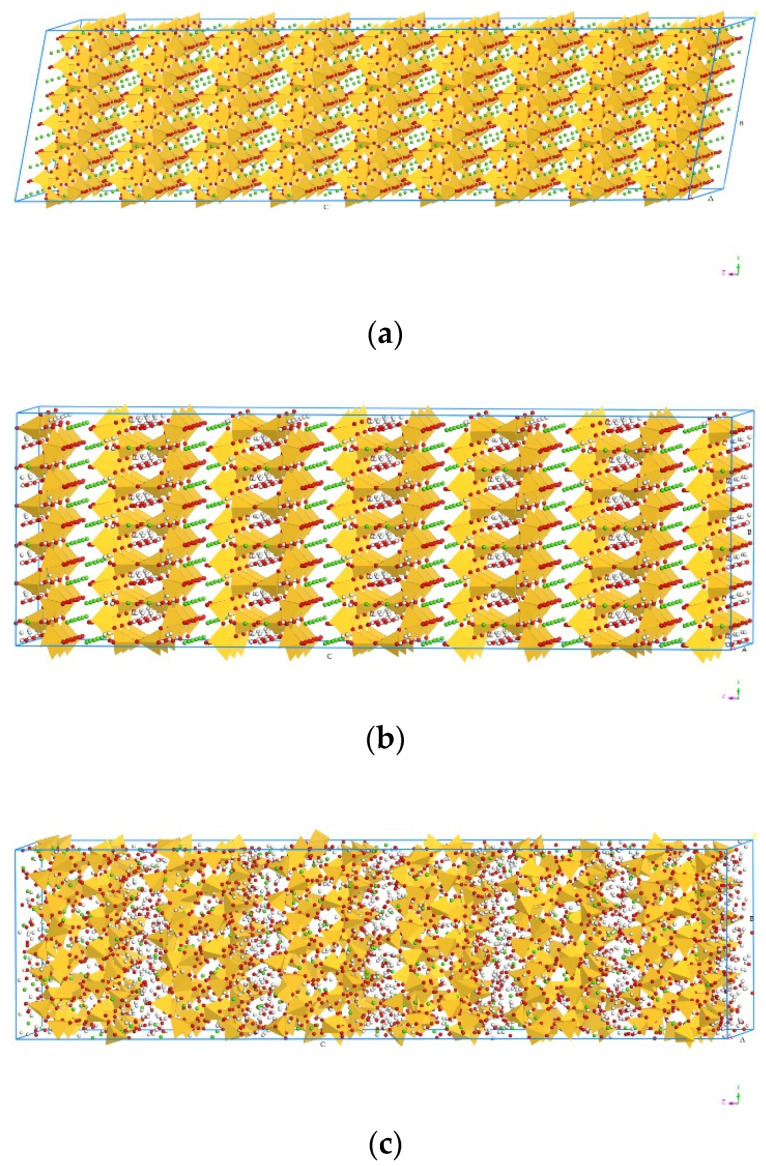
Molecular structure of Tobermorite: (**a**) Tobermorite 9 Å, (**b**) Tobermorite 11 Å, (**c**) Tobermorite 14 Å (Color legend: hydrogen H (white); calcium Ca (green); oxygen O (red); silica Si (yellow polyhedral)).

C–S–H has been identified as a layered structure consisting of a calcium layer linked to a silicate chain, which follows the pattern of Dreierkette with a repeated unit in every three tetrahedrons [44]. Therefore, the structure of C–S–H contains a linear silicate chain in the form of Dreierkette, with a number of positive calcium ions arranged in each layer and a negative silicon chain in the middle, and the space between each layer contains water molecules and calcium ions [45]. Due to its similar structure to C–S–H hydrates, models of the Tobermorite family, including Tobermorite 9 Å (T9, Ca_5_Si_6_O_16_(OH)_2_), Tobermorite 11 Å (T11, Ca_4.5_Si_6_O_16_(OH)·5H_2_O) and Tobermorite 14 Å (T14, Ca_5_Si_6_O_16_(OH)_2_·7H_2_O), have been established to simulate the C–S–H [46,47], where 9, 11, and 14 Å represent the distance of space between layers caused by different hydration degrees. In this study, to minimize the influence of model size in the calculation process, as shown in Figure 3, the original supercell models of T9, T11 and T14, were set as 3a × 3b × 6c, 2a × 3b × 2c, and 3a × 3b × 3c, respectively. Considering the errors that may appear in a small system, the total number of the molecules in the three models was controlled at around 5000, which ensured that the system was large enough to be accurately simulated [48,49]. Moreover, to establish the interphase between the Tobermorite and the UF, the C–S–H sheet were cleaved along the [0 0 1] direction (*z*-direction), and the XY plane was set to contact the UF. By using the Build Vacuum, the Vacuum thickness was set to 0, so as to change the model into a periodic structure.

**Figure 4:**
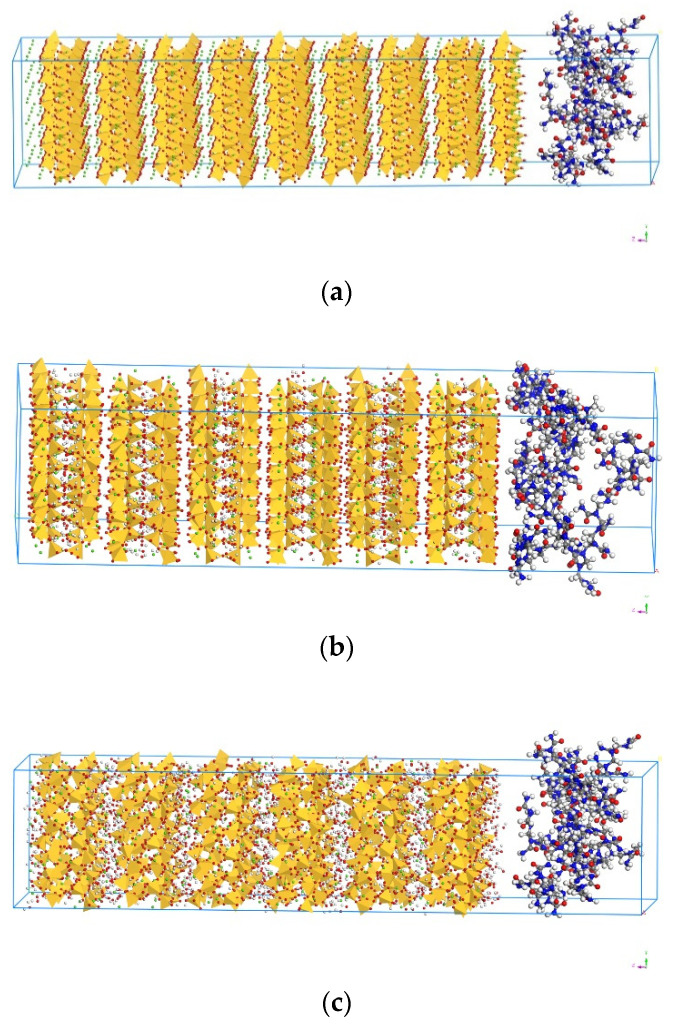
Molecular structure of Tobermorite-UF: (**a**) Molecular structure of T9-UF, (**b**) Molecular structure of T11-UF, (**c**) Molecular structure of T14-UF (Color legend: hydrogen H (white); calcium Ca (green); oxygen O (red); nitrogen N (blue) carbon C (grey); silica Si (yellow polyhedra)).

For building a composite model of C–S–H and UF, as shown in Figure 4a–c, the supercell of the composite structure with two layers of C–S–H and UF was applied, where the UF was set as layer 1 and Tobermorite was set as layer 2 (applied for T9, T11 and T14). All the calculations were then conducted based on these three composites structures as T9-UF, T11-UF and T14-UF. The optimisation of the model in this paper can be viewed in the Supplementary Materials.

### 2.3. Molecular Dynamics Simulation

The MD simulation was conducted via Materials Studio. The Tobermorite model was energy-minimized, which was performed using a smart algorithm. Ewald summation method, a summation algorithm which can be used to calculate the coulombic interactions between ions in systems with periodic boundary conditions [50,51], was employed to calculate the non-bonded energy including both electrostatic and van der Waals interactions, where the cut-off distance in all simulations was set as 12 Å. Then, the layer was built by following the procedure described in Section 2.2. In order to optimize the structure of UF, the Tobermorite was firstly constrained and the energy minimization was performed again by using the CVFF forcefield. Then, the constraints of the Tobermorite were removed and the energy minimization was performed again to balance and optimize the entire structure by COMPASS forcefield.

Since the weakest part of the layered structure model was identified as the *z*-direction [23], the stress–strain behavior of the combined composites model of Tobermorite and UF was simulated under a uniaxial tensile on the *z*-direction. The entire simulation was performed under NPT (isothermal–isobaric ensemble, N is the number of atoms, P is the pressure, T is the temperature) integration conditions by following the procedures: (1) calculate 1ns after setting the temperature of 300 K and the external pressure as 0; (2) after the stresses in all directions were calmed down, the model was stretched in the *z*-direction by inputting the stress parameters. The step size was set to 1 fs. During the simulation, the *x*- and *y*-directions remained relaxed. In order to analyze the simulation results, the calculation results were saved every 10,000 steps.

## 3. Results and Discussions

### 3.1. Mechanical Property

The mechanical properties, including bulk modulus, shear modulus and elastic modulus from all *x*-, *y*- and *z*-directions, of the three composites models are shown in Table 1. It can be seen from the table that the bulk modulus of three models was in the range between 26 and 60 GPa, with the highest value obtained in T9-UF. Similarly, the highest shear modulus of 24 GPa was observed in T9-UF as well, whereas that of the other two models was below 10 GPa. Moreover, regardless of direction, the Young’s modulus simulated in this study was in the range of 3–36 GPa, which was smaller than that obtained from the reported experiment data, 15–45 GPa [52,53]. Since both cementitious materials and microcapsules are complex materials containing different components, the difference between the experiment and MD simulation could be due to simplification by the MD simulation. For example: (1) in the experiment, the microcapsule incorporated concrete is a type of anisotropic material, however, the simulated elastic model was calculated only in three directions (*x*-, *y*-, and *z*-); (2) the microcapsule, including both the urea–formaldehyde shell and epoxy core, was used in the experiment, however, only the UF shell of the microcapsule was simulated in this study; (3) in the experiment, the elastic modulus varied, incorporating different numbers of microcapsules, however, the content of UF shell in the simulation was fixed; (4) in the experiment, in addition to C–S–H, other hydration products, such as ettringite and portlandite, may also exist in the cementitious matrix, however, the simulation was only conducted with C–S–H, the main hydration products of cement.

Moreover, it can be noticed that, among the Young’s modulus in three directions, the largest value of Young’s modulus was observed in the *y*-direction, which is similar to the simulation of ‘pure’ Tobermorite [54]. This could be due to the fact that the silicate chain stands steady in the *y*-direction, while the hydrogen bonding network breaks in the other two directions.

Noticeably, the Young’s modulus, bulk modulus and shear modulus simulated by these three models were smaller than those simulated by the model of ‘pure’ Tobermorite [27], suggesting that the addition of the UF-based microcapsule reduced the overall mechanical properties of cementitious materials. Those simulation results showed a good agreement with the experimental investigation that the elastic modulus of the concrete was decreased after adding microcapsules [52]. At the microscopic level, the decreased elastic modulus could be due the larger distance among the atoms of the Tobermorite–UF model than that of the ‘pure’ Tobermorite, which results in the lower value of the binding force between the layers. Moreover, the decrease in elastic modulus indicated that the model exhibited a better ductility with the greater elastic deformation, which will be discussed in Section 3.3 below.

### 3.2. Radial Distribution Function

**Figure 5:**
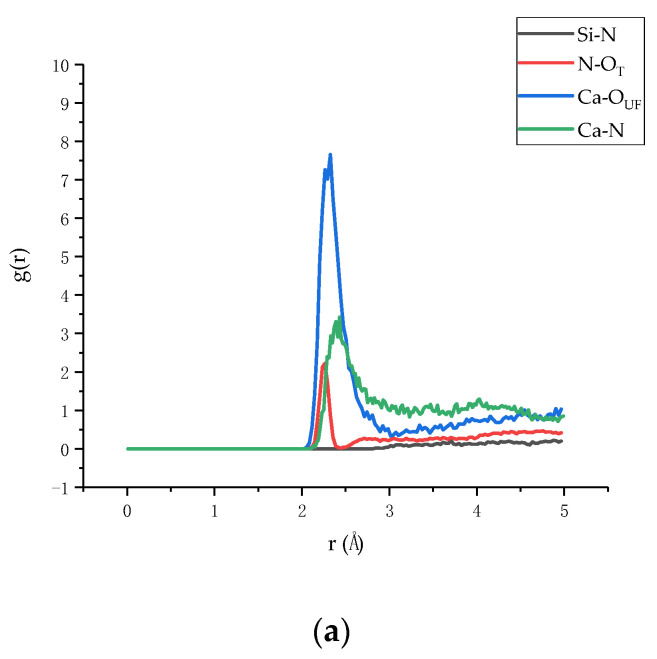

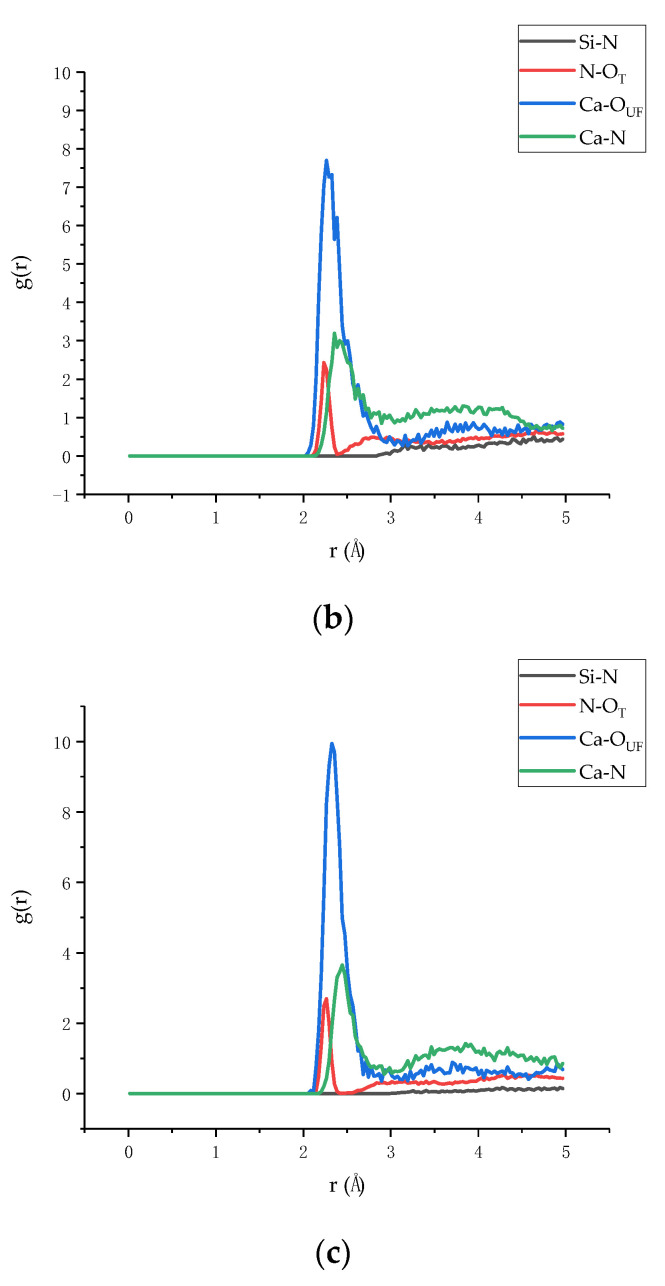
Radial distribution function (RDF): (**a**) RDF of T9-UF, (**b**) RDF of T11-UF, (**c**) RDF of T14-UF (The O_T_ and O_UF_ stand for the oxygen in Tobermorite and UF, respectively).

**Figure 6:**
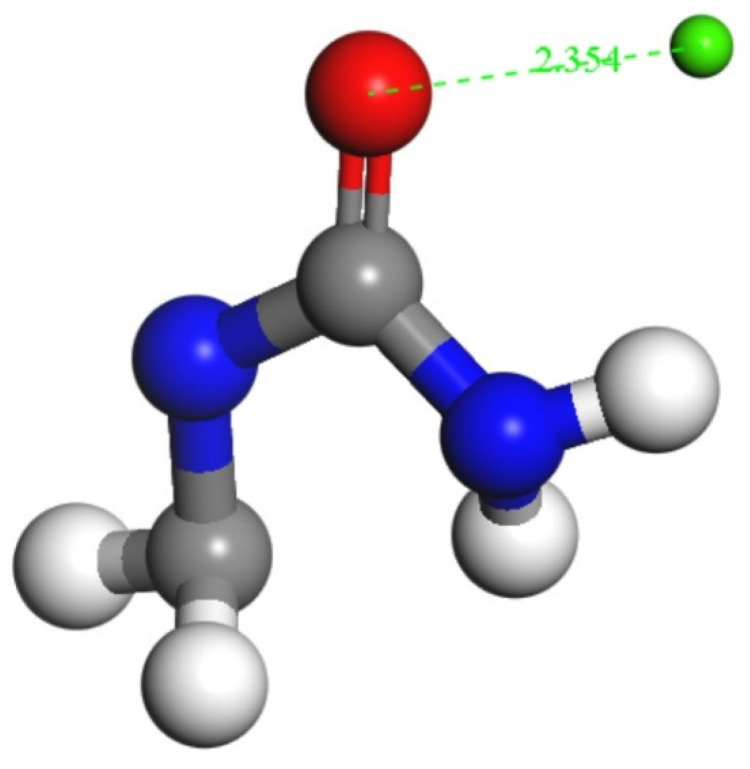
The reaction of UF and calcium ions (Color legend: hydrogen H (white); calcium Ca (green); oxygen O (red); nitrogen N (blue) carbon C (grey)).

In order to understand the binding strength of the interface between the Tobermorite and the UF, the atomic radial distribution function (RDF), a method to characterize the composite surface adsorption points of the two materials was calculated for describing the spatial correlation between atoms [55,56]. The RDFs of three composites models, in terms of T9-UF, T11-UF, and T14-UF, are presented in Figure 5. As shown in the figure, in general, the RDFs of the three models exhibited the same trend, indicating that the modes of action of atoms in the three models were consistent. However, it can be seen from the figure that the RDF of Ca–N, N–O_T_ and Ca–O_UF_ was different to that of Si–N. A peak in the RDF appeared in Ca–N, N–O_T_, Ca–O_UF_, while no peaks belonging to Si–N were observed. Moreover, comparing the three composite models, the peaks in Ca–N of T9–UF, T11–UF, T14–UF were located at 2.39 Å, 2.33 Å, 2.45 Å, while the peaks of N–O_T_ were at 2.27 Å, 2.24 Å, 2.27 Å, and Ca–O_UF_ at 2.33 Å, 2.27 Å, 2.33 Å. According to previous studies, calcium ions may bind to oxygen atoms if the distance between the oxygen atoms and the calcium ions is less than 2.5 [57,58]. It can be seen from Figure 5a–c that the first peak in RDF of Ca–O_UF_ in the three models was less than 2.5, and this result indicated the formation of a complex between UF and calcium ions (Figure 6).

The appeared sharp peaks indicated that the interface between the two materials was mainly due to the strong connection between Ca and N, N–O_T_ and Ca–O_UF_, while Si and N showed no interactions. Therefore, it can be deduced that the most important interaction is the electrostatic interaction between the positive charge Ca and the negative charge O. The Ca^2+^ ion exhibits a bridging effect at the interface [55], which enhanced the electrostatic effect on the interface binding energy. Therefore, the simulation results indicated that Ca^2+^, O^2−^ and N^−^ ions mainly play a bonding role at the interface.

### 3.3. Stress–Strain Curve

**Figure 7:**
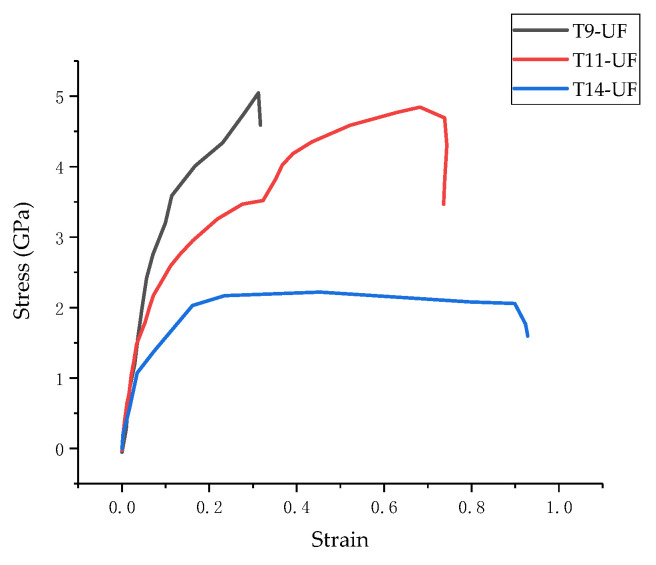
Stress–strain curves of T9-UF, T11-UF, T14-UF.

**Figure 8:**
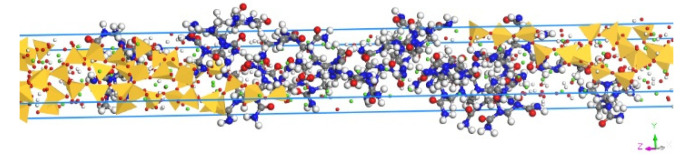
Snapshot of model stretch (Color legend: hydrogen H (white); calcium Ca (green); oxygen O (red); silica Si (yellow polyhedra); nitrogen N (blue) carbon C (grey)).

The stress–strain curves of the three models in *z*-direction are plotted in Figure 7. It can be seen from the figure that there are three stages of stress variation in the tensile process: (1) the stress increases linearly in the elastic stage; (2) the stress increases slowly in the yield stage; (3) the stress reduces in the failure stage. Moreover, based on the stress–strain curve, the tensile strengths of the three composites models of T9-UF, T11-UF and T14-UF, were 5.05, 4.84 and 2.22 GPa, respectively. Clearly, the strength of T14-UF was obviously smaller than that of the other two models. This could be because the layer spacing of T14-UF model was larger than that of the other two models, which therefore showed a weak connection between intermediate water and Ca^2+^ ion. Compared with the results generated from the model of pure Tobermorite, which were reported as 0.87, 0.93 and 1.01 GPa in the literature [23], the simulated strengths of the three composite models were larger, which indicated that the tensile strength in the *z*-direction increased when the UF microcapsule was added. The results were similar to the finding with the addition of graphene in cementitious matrix [59]. The increased tensile strength in the Tobermorite–UF model can be explained by the strong binding strength between Tobermorite and UF. As reported in Section 3.2, the Ca, O in Tobermorite and N and O in UF exhibited strong connection, which ensured a stronger connection between the UF and the Tobermorite. Therefore, when the model was uniaxially stretched in the *z*-direction, UF was effectively connected to Tobermorite. As shown in Figure 8, when Tobermorite fractures between layers (the left and right side of the UF) occurred, the UF can reduce the crack propagation, and thus increase the strength of the model.

Moreover, as shown in the Figure 7, the failure strains of the three models were 0.31, 0.68 and 0.45 respectively. Compared to the results from the model of ‘pure’ Tobermorite 11 Å of 0.2 [23], higher values were obtained in this study. This could due to the strong connection between Tobermorite and UF as proved in Section 3.2. When cracks began to appear between layers of Tobermorite, UF connected with the Tobermorite, thus delaying the propagation of cracks. Moreover, since UF is a chain structure, which can be expanded through stretching, compared to ‘pure’ Tobermorite, the entire composite model then becomes more malleable. The experiment shows that the ultimate strain of concrete was increased after adding UF-based microcapsules, which proved the feasibility of the results in this simulation [12]. The strain energy can be obtained by integrating the stress–strain curve by following the procedures proposed by Mohiuddin et al. [60]. According to the curves in Figure 7, the strain energies before the fracture of T9-UF, T11-UF and T14-UF were 1.09, 2.46 and 0.85 GPa, respectively. Unfortunately, the strain energy of ‘pure’ Tobermorite was missing, since it has been reported that higher elastic modulus results in a lower strain energy under the same stress [61]. As discussed in Section 3.1, the elastic modulus of Tobermorite–UF models was less than that of the ‘pure’ Tobermorite model. Therefore, it can be deducted that, under the same stress, the strain energy of the Tobermorite–UF model would be greater than that of ‘pure’ Tobermorite, and the ductility of the Tobermorite–UF model would be better than that of ‘pure’ Tobermorite.

### 3.4. Binding Energy

The binding strength of the interface between Tobermorite and UF can be evaluated by calculating the adhesion energy [62,63,64]. Therefore, in this study, the binding energy obtained as the opposite of the adhesion energy between the Tobermorite and the UF was calculated by following Equation (1) below [65]

(1) $$E_{b}=-E_{I}=-[E_{Total}-\left(E_{T}+E_{UF}\right)$$

where $E_{b}$ stands for the binding energy, $E_{I}$ stands for the interaction energy between the Tobermorite and the UF, $E_{total}$ for the total energy of the whole model, and $E_{UF}$ for the energy of the Tobermorite and UF.

The binding energy of three composite models is shown in Table 2. The binding energy of the three composite models was listed in the descending sequence of T9-UF, T11-UF and T14-UF. Moreover, as shown in the table, the binding energy of the T11-UF model was 2179 kcal/mol, which was similar to that of the T11- graphene oxide (GO) model (2338 kcal/mol) [66]. Since the greater the binding energy, the stronger the binding between the Tobermorite and the UF, the highest binding energy in T14-UF suggested that the easiest and closest interaction was obtained between T14 and the UF. As shown in Figure 5, the peak in the T14-UF model was higher and sharper than the other two models, which indicated a strong order and a tight relationship between atoms. This is consistent with the conclusion obtained here that the highest binding energy was observed in the T14-UF model. As discussed in Section 3.2, such a high binding energy between the Tobermorite and the UF could be mainly formed by the electrostatic interaction between Ca^2+^, O^2−^ and N^−^ ions.

In sum, it can be indicated that Tobermorite can be connected with the UF shell, which can improve the strength of the composite. Moreover, the results suggested that the embedded microcapsule with the UF resin shell can attach to the hardened cementitious matrix, which cannot easily peel off and lose the self-healing performance.

## 4. Conclusions

In this paper, the mechanical properties and bonding behavior, including the interaction, binding energy and stress–strain curves, of three Tobermorite–UF resin composite models were studied by MD simulation to illustrate the performance of UF/Epoxy microcapsule in cementitious material. The results showed that there was a high binding energy between Tobermorite and UF, which was highly dependent on the strong interactions of Ca, N and O between Tobermorite and UF interfaces. The stress and strain curves by uniaxial tension of T9-UF, T11-UF and T14-UF revealed that the strength and failure strain of Tobermorite–UF were high, indicating a better ductility of the Tobermorite–UF models. The large binding energy between the Tobermorite and UF suggested a good compatibility of the UF/Epoxy microcapsule, which can be strongly adhered with the cementitious matrix. The results reveal the effectiveness of the microcapsule-based self-healing cementitious system.

## Figures and Tables

**Table 1:** The bulk modulus, shear modulus and elastic modulus of the three models.

Model	Bulk Modulus (GPa)	Shear Modulus (GPa)	Young’s Modulus (GPa)		
			*x*	*y*	*z*
T9-UF	59.163	23.9466	11.4652	17.6246	3.3996
T11-UF	28.5138	5.7435	9.7172	24.4057	11.6104
T14-UF	26.4379	8.6146	24.1895	36.9385	20.187

**Table 2:** The binding energy of three models.

Model	*E_b_* (kcal/mol)
T9-UF	1394
T11-UF	2179
T14-UF	3334

## Supplementary Materials

The following are available online at https://www.mdpi.com/1996-1944/13/18/4054/s1, Figure S1. The energy curve of the first step. Figure S2. The energy curve of the second step. Figure S3. The energy curve of the third step.
