# Supplementary material for: Molecular Dynamics Study on Mechanical Properties of Interface between Urea-Formaldehyde Resin and Calcium-Silicate-Hydrates
Source: Materials (Basel). 2020 Sep 12;13(18):4054. doi: 10.3390/ma13184054 (PMC7558882; doi:10.3390/ma13184054)
Supplement: Supplementary file 1 [file materials-13-04054-s001.pdf]

materials

# Supplementary Materials: Molecular Dynamics Study on Mechanical Properties of Interface between Urea-Formaldehyde Resin and Calcium-Silicate-Hydrates

**Xianfeng Wang [1], Wei Xie [1], Taoran Li [1], Jun Ren [1,2,*], Jihua Zhu [1,*], Ningxu Han [1] and Feng Xing [1]**

[1] Guangdong Provincial Key Laboratory of Durability for Marine Civil Engineering, College of Civil and Transportation Engineering, Shenzhen University, Shenzhen 518060, China; xfw@szu.edu.cn (X.W.); xw13671400570@163.com (W.X.); litaoran2016@email.szu.edu.cn (T.L.); nxhan@szu.edu.cn (N.H.); xingf@szu.edu.cn (F.X.)

[2] Harbin Institute of Technology, School of Science, Shenzhen 518055, China

* Correspondance: renjunking@aliyun.com (J.R.); zhujh@szu.edu.cn (J.Z)

## 1. Model Optimization

The optimization of the model in this paper was divided into three steps. The first step was to optimize the Tobermorite model. The Tobermorite model was energy minimized by COMPASS force field, which was performed by using the smart algorithm. Ewald summation method was utilized to calculate the non-bonded energy including both electrostatic and van der Waals interactions, and the cut-off distance in all simulations was set as 12 Å. The second step was to optimize the urea-formaldehyde resin. Tobermorite was fixed and optimized using CVFF force field. The rest of the settings were the same as in step 1. The third step was to optimize the whole composite model. Remove the constraint of Tobermorite and optimize the entire model using COMPASS force field. The rest of the settings were the same as in step 1. The energy diagrams of the optimization results of the three steps are shown in Figure S1–S3. It can be seen from the figure that after each step of optimization, the energy tended to be stable and reached the minimum value, indicating the completion of structural optimization.

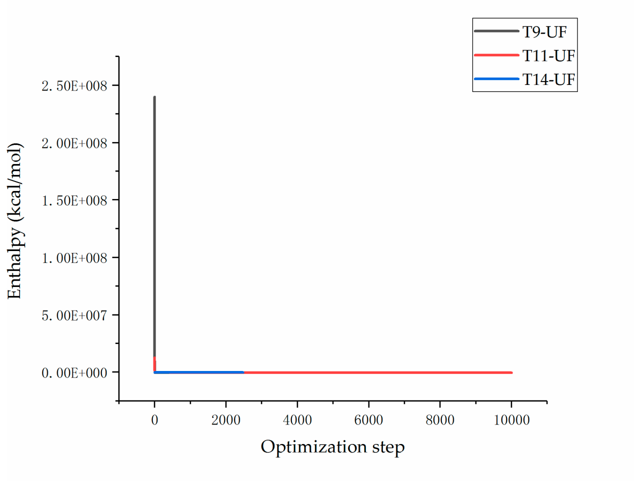


**Figure S1.** The energy curve of the first step.

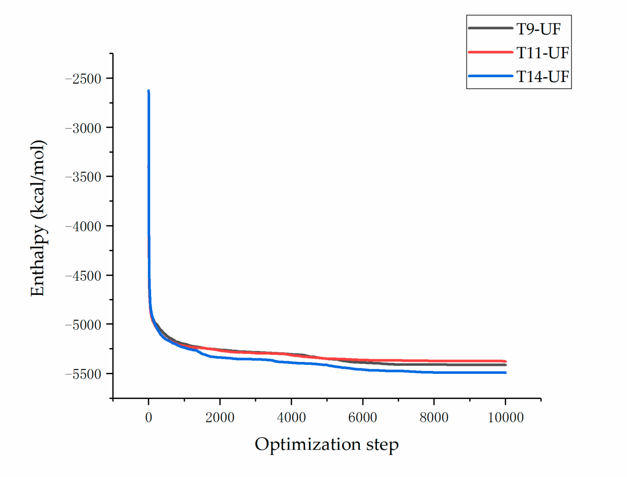


**Figure S2.** The energy curve of the second step.

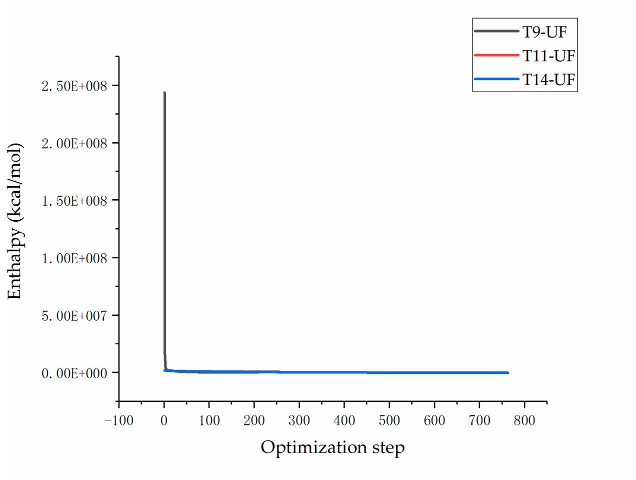


**Figure S3.** The energy curve of the third step.

© 2020 by the authors. Submitted for possible open access publication under the terms and conditions of the Creative Commons Attribution (CC BY) license (http://creativecommons.org/licenses/by/4.0/).
